# Is the Presence of 2 Renal Allograft Arteries Associated with Adverse Outcomes in Live Donor Kidney Transplantation?

**DOI:** 10.5152/eurasianjmed.2022.0259

**Published:** 2023-02-01

**Authors:** Eryiğit Eren, Mehmet Tokaç, Bora Uslu, Taylan Şahin, Talar Vartanoğlu Aktokmakyan, Ayhan Dinçkan

**Affiliations:** 1 Department of General Surgery, Istinye University Training and Research Hospital, İstanbul, Turkey; 2 Department of Internal Medicine, Division of Nephrology, İstinye University Training and Research Hospital, İstanbul, Turkey; 3 Department of Anesthesiology, İstinye University Training and Research Hospital, İstanbul, Turkey

**Keywords:** Renal allograft artery, double, kidney transplantation, live donor, laparoscopic

## Abstract

**Objective:**

Although it was postulated that renal grafts with multiple arteries could lead to unfavorable recipient outcomes, this subject remains controversial. This study aimed to compare the outcomes of recipients receiving renal allografts with a single artery with those receiving renal grafts with two arteries.

**Materials and Methods:**

Adult patients who received live donor kidney transplantation in our center between January 2020 and October 2021 were included. Data including age, gender, body mass index, renal allograft side, pre-kidney transplantation dialysis status, human leukocyte antigen mismatch number, warm ischemia time, the number of renal allograft arteries (single/double), complications, duration of hospitalization, postoperative creatinine levels, glomerular filtration rates, early graft rejection, graft loss, and mortality were collected. Subsequently, patients who received single-artery renal allografts were compared with those who received double-artery renal allografts.

**Results:**

Overall, 139 recipients were included. The mean recipient age was 43.73 ± 13.03 (21-69). While 103 recipients were male, 36 were female. The comparison between the 2 groups revealed that mean ischemia time was significantly longer in the double-artery than in the single-artery group (48.0 vs. 31.2 minutes) (*P* = .00). In addition, the single-artery group had significantly lower postoperative day 1 and day 30 mean serum creatinine levels. Also, the mean postoperative day 1 glomerular filtration rates were significantly higher in the single-artery group than in the double-artery group. However, the 2 groups were similar concerning the glomerular filtration rates measured at other times. On the other hand, there was no difference between the 2 groups regarding duration of hospitalization, surgical complication, early graft rejection, graft loss, and mortality rates.

**Conclusion:**

The presence of 2 renal allograft arteries does not have adverse effects on the postoperative parameters of the kidney transplantation recipients, including graft function, duration of hospitalization, surgical complication, early graft rejection, graft loss, and mortality rates.

**Main Points**

- The presence of 2 renal allograft arteries does not have adverse effects on recipient graft function.
- The early graft rejection, graft loss, and mortality rates are not affected by the presence of 2 renal allograft arteries.
- The presence of 2 renal allograft arteries does not prolong the duration of hospitalization and increase the surgical complication rates.

## Introduction

Kidney transplantation (KT) is the gold standard treatment method for end-stage renal disease patients.^1^ The kidney can be donated by deceased or living donors, and non-invasive donor nephrectomy methods have become popular for live kidney donation in recent decades. Currently, live donor nephrectomy procedures can be performed by open, laparoscopic, or robotic methods. In 1995, the first laparoscopic donor nephrectomy (LDN) was performed by Ratner et al.^2^ Since then, numerous kidney transplant centers have adopted the LDN approach due to its advantages, such as fewer complications and shorter hospitalization.^3^

It is known that the outcomes of live donor KT are more favorable than those of deceased donor KT. Therefore, due to the increased demand for KT, every effort is made by transplant surgeons to utilize live donor kidneys despite variations such as multiple renal arteries.^4,5^ It was previously reported that the rate of multiple renal arteries ranged between 18% and 30%, and approximately 15% of the donor candidates had multiple arteries in both kidneys.^6^ Although it was postulated that renal allografts with multiple arteries could lead to unfavorable recipient outcomes, this subject remains controversial.^7,8^ Therefore, this study aimed to compare the outcomes of recipients receiving renal allografts with a single artery with those receiving renal grafts with two arteries.

## Materials and Methods

Adult (i.e., age 18 or older) patients who received live donor KT in our center between January 2020 and October 2021 constituted the target population of this study. It was approved by the ethical review committee of İstinye University (22.11.2022/22-140). All patients gave written consent before enrollment in the study. Patient data including age, gender, recipient body mass index (BMI), donor BMI, renal allograft side (left/right), pre-KT dialysis status (preemptive/hemodialysis/peritoneal dialysis), and human leukocyte antigen mismatch number were retrieved from the electronic patient folders. Patients who had incomplete data and were followed for less than 1 year were excluded. The collected data also included warm ischemia time, the number of renal allograft arteries (single/double), surgical and post-surgical complications, postoperative serum creatinine (Cr) levels (day 1, day 7, day 30, 6 months, and 12 months), glomerular filtration rates (GFRs) (day 1, 6 months, and 12 months), duration of hospitalization, early graft rejection, graft loss, and mortality. Patients who received single-artery renal allografts were compared with those who received double-artery renal allografts regarding the data parameters to assess the impact of multiple arteries on recipient outcomes.

All recipients received a renal allograft procured by an LDN procedure. All donor surgeries were performed by a pure laparoscopic technique, including a transperitoneal approach and a Pfannenstiel incision. The primary surgeon who performed all LDN and KT procedures decided on the renal allograft side. The left kidney was preferred in all cases with normal renovascular anatomy. In patients with renal cysts or clinically insignificant renal anomalies (calcifications or millimetric stones), the kidney with these anomalies was transplanted to protect the donor from risks. Double-artery presence was not considered a contraindication for LDN, particularly in the setting of donors with multiple arteries in both kidneys. All KT procedures were performed via an open extraperitoneal approach. All vascular anastomoses were end-to-side vascular anastomoses between renal allograft vessels and external iliac vein and artery. All patients underwent ureter–urinary bladder anastomosis with the extravesical Lich–Gregoir procedure, during which a double J stent was placed.

Warm ischemia time was calculated as the sum of warm ischemia time at the donor and anastomosis time at the recipient sides. The glomerular filtration rate was estimated using the 4-variable Modification of Diet in Renal Disease study equation. Early graft rejection was defined as rejection proven by a graft biopsy during the first 3-month period after KT. Double-artery renal allografts were defined as renal allografts necessitating the performance of 2 separate artery anastomoses.

### Statistical Analysis

Data analysis was performed using IBM Statistical Package for Social Sciences Statistics version 25.0 software (IBM SPSS Corp., Armonk, NY, USA). The normal distribution of the data was tested by the Shapiro–Wilk test. Categorical data were expressed as numbers (n) and percentages (%), while quantitative data were given as means ± SDs. The Mann–Whitney *U*-test was used to compare non-normally distributed variables. Pearson’s *χ* ^2^ test was used in categorical data analysis unless otherwise stated. The 2 × 2 contingency tables were used to compare the categorical variables; Fisher’s exact test was performed when 1 or more cells had an expected frequency of 5 or less. A *P*-value less than .05 was considered statistically significant.

## Results

In total, 139 recipients were included (Table 1). The sociodemographic characteristics of the study population are displayed in Table 1. The mean recipient age was 43.73 ± 13.03 (21-69). While 103 recipients were male, 36 were female. The most common primary diagnoses were hypertension and diabetes mellitus.

The mean recipient and donor BMI were 25.88 and 29.45 kg/m^2^, respectively. Ninety-nine recipients received a left kidney, while 40 patients received a right donor kidney. While 62 patients underwent preemptive KT, 77 recipients were on hemodialysis at the time of KT. No recipients were on peritoneal dialysis.

The comparison regarding preoperative characteristics and operative parameters revealed statistically significant differences in donor gender distribution, renal allograft side, and ischemia time (Table 2). The mean ischemia time was significantly longer in the double-artery than in the single-artery group (48.0 vs. 31.2 minutes) (*P* = .00). The comparison of the 2 groups concerning graft function showed that the single-artery group had significantly lower postoperative day 1 and day 30 mean serum Cr levels (Table 3). The mean postoperative day 1 GFR was significantly higher in the single-artery group than in the double-artery group. However, the 2 groups were similar concerning the GFR measured at other times.

Overall, 5 patients had postoperative surgical complications. Among these patients, 4 (2 from the single-artery and 2 from the double-artery group) had retroperitoneal bleeding. Two of these patients were treated conservatively, while the other 2 (1 from the single-artery and 1 from the double-artery group) necessitated emergent surgical exploration. In addition, 1 patient from the double-artery group had a urine leak which required a revision of the ureteroneocystostomy. This patient received a renal allograft with a double artery.

In our cohort, 5 (3.6%) patients lost their renal grafts, and all graft failure cases were due to rejection. One of these patients died due to the new-type coronavirus infection. There was no other mortality in our study. In addition, there was no difference between the 2 groups regarding the duration of hospitalization, surgical complication, early graft rejection, graft loss, and mortality rates.

## Discussion

Since LDN is associated with relatively few complications and fast recovery, it is the preferred donor nephrectomy method in many transplant centers.^9,10^ The left kidney is usually selected due to the longer renal vein on the left side than on the right.^10^ Regardless of the renal allograft side, multiple renal arteries can be present in a donor as an anatomical variation. Although there is a debate in the literature regarding the use of renal allografts with multiple renal arteries for live donor KT, it is a fact that there is an increasing demand for the use of these organs.^4,5,11^ Our study compared patients who received single-artery renal allografts with those who received double-artery renal allografts.

Similarly, Desai et al^5^ compared recipient outcomes in patients with single renal allograft arteries with those who had multiple arteries. Again, all patients underwent LDN. This study which included 303 patients concluded that multiple arteries did not impact graft function at 1 month and 1 year. Our results are in line with this study.

Fitzpatrick and coworkers^12^ worked on 465 patients who underwent LDN. While 359 patients received a renal allograft with a single artery, 106 received grafts with multiple arteries. The comparison regarding vascular and ureteral complications did not reveal a significant difference. Two recipient groups were also similar regarding acute rejection and 1-year, 5-year, and 10-year graft survival rates. These authors concluded that the presence of multiple renal allograft arteries did not constitute a contraindication for renal transplantation. In our study, the renal graft functions were followed for a year. However, our results are similar to those of Fitzpatrick et al.^12^

Keller et al^13^ compared the procurement of the kidneys with a single artery with those with multiple arteries concerning parameters such as ischemia time, estimated blood loss, duration of hospital stay, and delayed graft function. These researchers worked on 230 patients who underwent LDN. Although they found that warm ischemia time was significantly longer in recipients receiving renal allografts with multiple arteries, they noted that the presence of multiple arteries did not affect the other outcomes. In line with this study, we found that the mean warm ischemia time was significantly longer in the double-artery group than in the single-artery group, and the presence of a double-artery did not impact transplant outcomes. As described in the “Methods” section, we defined the warm ischemia time as the sum of warm ischemia time at the donor and anastomosis time at the recipient sides. Unfortunately, we did not have separate warm ischemia time recordings for the donor and recipients (i.e., anastomosis time) in our database. However, we believe that the difference in the warm ischemia times of our study groups is caused by the difference in the anastomosis times since the anastomosis of 2 arteries takes significantly longer than the anastomosis of a single artery.

Meyer and coworkers^14^ evaluated their experience with LDN in patients with multiple renal arteries and compared their outcomes with those who received allografts with a single artery. They included 130 patients, 22 of whom received allografts with multiple arteries. They reported no significant difference between the 2 groups regarding graft function. Therefore, they concluded that the presence of multiple renal allograft arteries was not a contraindication for renal transplantation.

In their study, including 97 live donor kidney transplants, Mahajan et al^15^ compared the clinical outcomes of the recipients who received allografts with a single renal artery with those who had multiple arteries. All KT surgeries were performed by an open approach. These authors noted that the ischemia time was significantly higher in the latter group than in the former. Also, they did not find a difference between the 2 groups regarding 1-year graft and patient survival. They concluded that live donor KT was feasible and safe in patients receiving renal allografts with multiple arteries. Our results align with those of Meyer et al^14^ and Mahajan et al.^15^

Cooper et al^16^ investigated the outcomes of KT patients who received renal allografts with single or multiple arteries. They analyzed the data of 147 patients and compared 2 patient groups regarding graft failure and complication rates and patient and graft survival. These comparisons did not reveal statistical significance. Therefore, these researchers concluded that the presence of multiple renal allograft arteries did not predict graft failure, and it did not impact the analyzed transplant outcomes. Similarly, Tyson et al^17^ compared the outcomes of 393 patients who received a renal allograft with a single artery with 117 patients who received grafts with multiple arteries. The comparison concerning complication, acute rejection rates, and graft and patient survival revealed that multiple renal allograft arteries did not adversely affect graft and patient survival. Our findings are in line with these studies.

Our study has some limitations that must be considered while evaluating its findings. First, it is a retrospective study. Second, the sample size is relatively small. Third, the follow-up period is relatively short. However, while most studies in the literature included patients with 2 or 3 renal allograft arteries in the same group, only patients with a single renal allograft artery or those with double arteries were included in our cohort. Therefore, this study design can be considered as a strength of our study.

## Conclusion

Despite the limitations mentioned above, we conclude that the presence of a double renal allograft artery does not have adverse effects on the postoperative parameters of the KT recipients, including graft function, duration of hospitalization, surgical complication, early graft rejection, graft loss, and mortality rates.

## Figures and Tables

**Table 1.** Sociodemographic Characteristics of the Renal Transplant Patients (n = 139)

Variable	Category	Mean or n	SD or %
Age (years), mean (SD)	Recipient	43.73	13.03
	Donor	41.18	13.08
Gender	Female	36	25.9%
	Male	103	74.1%
Recipient BMI		25.88	4.85
Donor BMI		29.45	5.20
Side	Left	99	71.2%
	Right	40	28.8%
Ischemia time		34.71	8.41
Dialysis status	Preemptive	62	44.6%
	HD	77	55.4%
HLA mismatch number		2.95	2.00

BMI, body mass index; HD, hemodialysis; HLA, human leukocyte antigen.

**Table 2.** Comparison Regarding Preoperative Characteristics and Operative Parameters (n = 139)

Variables	Single Artery, n = 110 (79.1%)	Double Artery, n = 29 (20.9%)	*P*
Recipient age (years)	43.45 (13.06)	44.79 (13.06)	.624
Donor age (years)	47.67 (13.61)	45.31 (10.81)	.723
Recipient gender			.054^*^
Female	24 (17.3)	12 (8.6)	
Male	86 (61.9)	17 (12.2)	
Donor gender			.005^*^
Female	71 (51.1)	10 (7.2)	
Male	39 (28.1)	19 (13.7)	
Recipient BMI	26.10 (4.91)	25.00 (4.60)	.273
Donor BMI	29.24 (4.89)	30.23 (6.25)	.273
Side			.039^*^
Left	83 (59.7)	16 (11.5)	
Right	27 (19.4)	13 (9.4)	
Ischemia time	31.20 (4.72)	48.00 (5.56)	.000
Dialysis status			.679^*^
Preemptive	48 (34.5)	14 (10.1)	
Hemodialysis	62 (44.6)	15 (10.8)	
HLA mismatch number	2.91 (1.99)	3.10 (2.08)	.645

^*^Fisher’s exact test result.

BMI, body mass index; HLA, human leukocyte antigen.

**Table 3.** Comparison Regarding Postoperative Outcomes

Variables	Single Artery (n = 110)	Double Artery (n = 29)	*P*
PO day 1 Cr, mean (SD)	2.28 (1.14)	2.85 (1.15)	.012
PO day 7 Cr, mean (SD)	1.00 (0.39)	2.00 (9.34)	.062
PO day 30 Cr, mean (SD)	1.03 (0.24)	2.12 (9.04)	.003
PO 6-month Cr, mean (SD)	1.10 (0.30)	1.27 (0.45)	.060
PO 12-month Cr, mean (SD)	1.22 (0.48)	1.31 (0.50)	.160
PO day 1 GFR, mean (SD)	105.92 (145.34)	77.39 (91.62)	.012
PO 6-month GFR, mean (SD)	76.35 (28.20)	67.84 (22.42)	.231
PO 12-month GFR, mean (SD)	67.18 (21.49)	69.91 (24.23)	.567
Duration of hospitalization (days)	7.21 (3.94)	7.41 (3.79)	.770
Surgical complication, n (%)	3 (2.2)	2 (1.4)	.279^*^
Early graft rejection, n (%)	36 (32.7)	4 (2.9)	.064^*^
Graft loss, n (%)	4 (2.9)	1 (0.7)	.961
Mortality, n (%)	1 (0.7)	0 (0)	.606

^*^Fisher’s exact test result.

Cr, creatinine; GFR, glomerular filtration rate; PO, postoperative.
